# Successful methotrexate rescue following antibody-mediated infliximab failure for treatment-refractory hidradenitis suppurativa

**DOI:** 10.1016/j.jdcr.2026.02.049

**Published:** 2026-04-02

**Authors:** Richard Rookwood, Zahidul Islam, Nicole Schiraldi, Sarah Romanelli, Lana Salloum, Kristina Campton, David Ciocon

**Affiliations:** a Division of Dermatology, Department of Medicine, Montefiore Medical Center, Albert Einstein College of Medicine, Bronx, New York; b Department of Dermatology, New York Medical College, Valhalla, New York

**Keywords:** anti-drug antibodies, biologic immunogenicity, hidradenitis suppurativa, infliximab, methotrexate, rescue therapy, treatment failure

## Introduction

Hidradenitis suppurativa (HS) is a chronic, relapsing inflammatory skin disease marked by recurrent nodules, abscesses, sinus tract formation, and inflammation of the apocrine glands. Adalimumab, secukinumab, and bimekizumab are currently the only biologic agents approved by the U.S. Food and Drug Administration (FDA) for moderate-to-severe HS. However, response rates remain modest. In the PIONEER I and II trials, adalimumab achieved HS clinical response (HiSCR) rates of only 41.8% and 58.9%.^1^ Similarly, in the phase 3 SUNSHINE and SUNRISE trials, secukinumab achieved HiSCR at week 16 in 42.3% and 46.1% of patients receiving monthly dosing.^2^ Most recently, in the phase 3 BE HEARD I and II trials, bimekizumab achieved HiSCR at week 16 in 48% and 52% of patients receiving biweekly dosing.^3^ Taken together, these findings suggest that currently approved biologic therapies inadequately control disease in approximately one third to one half of patients requiring treatment. Moreover, obesity and smoking, 2 common comorbidities in HS, have been associated with increased disease severity and reduced therapeutic response to biologics, posing a significant challenge in managing severe HS.^4^^,^^5^

Intravenous (IV) infliximab (IFX) is frequently used off-label in patients with HS who have failed to respond to other biologic therapies. Advantages of IFX include its weight-based dosing and flexible infusion intervals. Although not currently FDA-approved for HS, data over the past 2 decades have demonstrated symptomatic improvement in patients receiving IFX for concurrent inflammatory bowel disease (IBD) or rheumatoid arthritis (RA).^6^^,^^7^ More recent studies have reported clinical response rates of up to 80% in patients treated with various IFX dosing regimens for moderate-to-severe HS.^8^^,^^9^ Despite these promising outcomes, long-term use is associated with an increased risk of developing anti-drug antibodies (ADAs), which may compromise therapeutic efficacy.

Methotrexate (MTX), an antimetabolite and immunomodulatory agent, is known to reduce the likelihood of anti-IFX antibody formation when used concomitantly with infliximab. In patients with IBD and RA who develop anti-IFX Abs, MTX has also been used as rescue therapy, leading to restoration of clinical response.^10^^,^^11^ However, there is a notable lack of research evaluating MTX as rescue therapy in HS patients with documented anti-IFX Abs. Several studies have highlighted this gap and called for further investigation.^8^^,^^12^ We aim to contribute to this limited body of literature by presenting a case series of 4 patients with treatment-refractory HS who developed anti-IFX antibodies and were subsequently rescued with MTX therapy.

We conducted a single-center, retrospective chart review of patients 18 years and older with refractory hidradenitis suppurativa treated with intravenous infliximab who subsequently developed anti-drug antibodies and were initiated on methotrexate. Among 62 patients identified for initial review, 4 met criteria for inclusion based on confirmed ADA positivity and the availability of pharmacologic laboratory data both prior to and following MTX initiation. Data collected included demographics, comorbidities, concurrent medications, treatment timelines, MTX dosing, time to rescue, clinical markers of disease severity (Hidradenitis Suppurativa-Physician Global Assessment [HS-PGA] score and Numerical Rating Scale for pain [NRS-pain]), and pharmacologic laboratory results, including IFX trough levels and ADA titers measured using the Anser infliximab assay (Prometheus Laboratories). The following cases illustrate the clinical course and outcomes of 4 patients who met inclusion criteria.

## Case presentations

All 4 patients included in this case series had a longstanding history of moderate-to-severe HS with Hurley stage II or III disease affecting typical intertriginous areas. The cohort had a female predominance (3 of 4 patients), and ages ranged from 22 to 53 years. The average body mass index (BMI) was 34.38, reflecting the known association between HS and obesity. All patients were non-smokers or former smokers and had been on IFX therapy for a mean duration of 2.5 years (range: 0.5-4.7 years) prior to ADA detection. A summary of patient demographics and concomitant medications is provided in Table I.

**Table I:** Patient demographics and baseline characteristics

Case	Age	Sex	Hurley stage	Smoking status	BMI	Concomitant treatments
1	41	F	II	Nonsmoker	29.26	Fluoroquinolone/Rifampin/Metronidazole; Spironolactone
2	22	M	III	Former smoker	33.47	Fluoroquinolone/Rifampin/Metronidazole; Finasteride
3	43	F	III	Nonsmoker	36.48	Fluoroquinolone/Rifampin/Metronidazole; Finasteride; Intramuscular triamcinolone; Ertapenem
4	53	F	III	Former smoker	37.90	Fluoroquinolone/Rifampin/Metronidazole; Spironolactone

*BMI*, Body mass index.

Patients were receiving weight based IV IFX at doses ranging from 7.5 to 12.5 mg/kg with infusion intervals every 3 or 4 weeks, along with concomitant long-term antibiotics and/or hormonal therapies as part of their treatment regimen. These therapies remained stable during the period of ADA development but were insufficient to maintain disease control, prompting evaluation for secondary loss of response with assessment of IFX trough levels and ADAs in all cases. The median interval from IFX initiation to ADA detection was 870 days (IQR 263-1525), with concomitant trough levels ranging from <1.0 to 7.8 μg/mL. The addition of MTX was prompted by detection of ADAs in all 4 patients. Two patients started the medication as prescribed at the first clinic visit following ADA detection, while the other 2 experienced delays due to treatment hesitancy and inconsistent follow-up. In those delayed cases, the decision to start MTX was made at a later date, prompted by worsening disease activity.

MTX was introduced at weekly doses ranging from 2.5 to 15 mg. Duration on MTX varied from 3 to 12 months. All patients demonstrated eradication of ADAs following the addition of MTX, accompanied by increased serum IFX levels relative to baseline. Moreover, all patients experienced improvement in at least 1 domain of disease activity, most notably reductions in pain scores. Three out of four patients (Cases 1, 2, and 3) exhibited reductions in their maximum HS-PGA scores while receiving MTX therapy. No serious adverse events were reported while taking combination therapy. In all cases, MTX was taken concurrent with IFX infusions for the duration of follow-up. Three patients remained on combination therapy through their last recorded visit, after which they were lost to follow-up. In 1 patient, IFX infusions were discontinued due to insurance denial, prompting discontinuation of MTX as well. A detailed summary of treatment timelines, serologic findings, and clinical responses is provided in Table II.

**Table II:** Treatment course and clinical response to methotrexate rescue therapy

Case	IFX dose and frequency (mg/kg)	Time to ADA detection	Serum ADAs (mg/dL)	Time to MTX initiation	MTX dose (mg/wk)	Duration on MTX	Change in HS-PGA∗	Change in NRS-pain∗	Serum IFX (μg/mL)†	Total duration on IFX
1	7.5 q4wk	4 y 7 mo	5.2	1 mo	2.5	12 mo	Min: +1 Max: −1	Min: 0 Max: −5	2.9 → 34.0; +31.1	5 y 10 mo
2	10 q4wk	5 mo	39.5	27 mo	15	3 mo	Min: +1 Max: −1	Min: 0 Max: −3	<1.0 → >34.0; +34.0	2 y 11 mo
3	12.5 q3wk	1 y	6.5	5 mo	10	8 mo	Min: −1 Max: −2	Min: 0 Max: −5	7.8 → >34; +26.2	2 y 2 mo
4	7.5 q3wk	3 y 9 mo	5.9	1 mo	7.5	3 mo	Min: −1 Max: 0	Min: +2 Max: −1	<1.0 → 13.2; +13.2	4 y 2 mo

*ADA*, Anti-drug antibody; *HS-PGA*, hidradenitis suppurativa–Physician Global Assessment; *IFX*, infliximab; *max*, maximum; *min*, minimum; *mo*, months; *MTX*, methotrexate; *NRS-pain*, Numerical Rating Scale for pain; *q3wk*, every 3 weeks; *q4wk*, every 4 weeks; *y*, years.

∗ Minimum and maximum values represent the changes in lowest and highest scores recorded during MTX treatment compared to baseline, respectively.

† Serum IFX levels are reported as pre- and post-MTX values, along with the net change in trough concentration. Pre-MTX values correspond to the visit when ADAs were first detected, and post-MTX values reflect the most recent trough level measured during combination IFX and MTX therapy.

## Discussion

Infusion therapy is typically reserved for patients with HS who have severe, recalcitrant disease unresponsive to other treatments and the emergence of ADAs remains a major obstacle to sustained therapeutic efficacy with IFX. What’s more, recent data suggest that HS patients may be at increased risk for IFX immunogenicity relative to patients treated for other inflammatory conditions, highlighting the importance of early recognition and management of ADA-mediated treatment failure.^13^

Evidence-based decision-making is essential when initiating systemic immunosuppressive therapies, however the use of MTX as rescue therapy for patients with HS remains underexplored. This case series illustrates that the addition of MTX may successfully reverse ADA-mediated secondary IFX failure, leading to both serologic and clinical improvements in patients with moderate-to-severe HS.

Trough levels were subtherapeutic in 3 patients, consistent with reduced drug availability in the setting of immunogenicity, while 1 patient demonstrated therapeutic levels despite ADA positivity. Importantly, the eradication of detectable ADAs was accompanied by restoration of IFX trough levels in the 3 patients with initially subtherapeutic concentrations and a substantial increase in the remaining patient, suggesting that MTX not only neutralizes existing immunogenicity but may also prevent continued antibody formation during ongoing IFX therapy. Moreover, clinically meaningful reductions in HS-PGA scores and pain levels were observed despite heterogeneity in MTX dosing and timing, underscoring the adaptability of this approach to real-world practice.

Of note, MTX regimens varied between conservative single-dose prescribing and more standard immunomodulatory dosing. In this patient cohort, dose selection appeared to correspond with baseline disease severity at the time of ADA detection. More specifically, higher doses were generally prescribed in patients with higher HS-PGA scores and more frequent or prolonged flares. While this observation is descriptive in nature, it suggests that MTX dosing decisions in HS may be influenced by clinical disease severity at the time of ADA detection.

These findings align with prior experiences in patients with IBD and RA, where MTX co-therapy has demonstrated efficacy in mitigating biologic immunogenicity and suggest a role for MTX as a rescue strategy in patients with HS. Based on our findings, we recommend that MTX be initiated at the time of ADA detection, as this was our intention for all 4 patients, despite 2 ultimately delaying rescue therapy until a later date. With respect to monitoring practices, we suggest that serum IFX trough levels and ADA testing be checked in patients who demonstrate limited or reduced therapeutic efficacy. For many patients, this may present as an increased frequency or duration of flares, worsening HS-PGA scores despite previously controlled disease, or failure of IFX to adequately control disease. In these instances, testing should precede blind dose escalation to ensure that treatment decisions are informed by pharmacologic and immunogenic data. The absence of serious adverse events in our cohort further supports the tolerability of this combination therapy.

## Conclusions

While encouraging, our findings are limited by the descriptive design, small sample size, relatively short duration of follow-up, lack of recurrence data, and study cohort that is not statistically representative of the HS patient population at large. It is also possible that some of the observed improvements in clinical outcome measures are owed, at least in part, to natural fluctuations in disease status rather than treatment with MTX alone. Additionally, no validated therapeutic IFX trough levels have been established for HS, and thus, our interpretations were based on IBD literature, where concentrations <3-5 μg/mL are most often associated with reduced clinical response.^14^ Given these limitations, further prospective studies are needed to define the optimal dosing strategy, timing of initiation, and long-term outcomes of MTX rescue therapy in HS patients experiencing anti-TNF resistance. Future work should also investigate whether there is a role for MTX as prophylaxis, rather than solely as rescue therapy, against ADA development.

## Conflicts of interest

None disclosed.
